# Protective effects of bifidobacteria against enteropathogens

**DOI:** 10.1111/1751-7915.13460

**Published:** 2019-07-08

**Authors:** Wanping Aw, Shinji Fukuda

**Affiliations:** ^1^ Institute for Advanced Biosciences Keio University 246‐2 Mizukami, Kakuganji Tsuruoka Yamagata 997‐0052 Japan; ^2^ Intestinal Microbiota Project Kanagawa Institute of Industrial Science and Technology 3‐25‐13 Tonomachi, Kawasaki‐ku Kawasaki Kanagawa 210‐0821 Japan; ^3^ Transborder Medical Research Center University of Tsukuba 1‐1‐1 Tennodai Tsukuba Ibaraki 305‐8575 Japan; ^4^ PRESTO Japan Science and Technology Agency 4‐1‐8 Honcho Kawaguchi Saitama 332‐0012 Japan

## Abstract

Recent major advances in metagenomics and metabolomics technologies have enabled us to collect more data on the gut microbiome and metabolome to evaluate its influence on host health. In this short opinion article, we have chosen to focus on summarizing the protective mechanisms of bifidobacteria, a highly regarded probiotic, and it's metabolite: acetate; against enteropathogens, specifically in the *E. coli* O157:H7 mice model. We advocate for using a novel approach metabologenomics, which is an integration of metagenomic and metabolomic information on a systems biology‐wide approach to better understand this interplay between gut microbiome and host metabolism.

## Gut microbiota and its metabolites

There are 40 trillions of bacteria residing in the human gut. The gut microbiota greatly influences human physiology, immunology and nutrition (Bäckhed *et al*., 2005; Kau *et al*., 2011). Imbalances in the gut environment, also known as dysbiosis, are related to various disorders such as metabolic disorders, inflammatory bowel disease and colon cancer (Frank *et al*., 2007; Turnbaugh *et al*., 2008; Wen *et al*., 2008; Wu *et al*., 2009). Therefore, in order to maintain good health, controlling gut environment balance may be effective to maintain a good health status in humans. Recent studies have also found that the gut microbiota produces a large variety of metabolites such as short‐chain fatty acids (SCFAs) (Flint *et al*., 2012; den Besten *et al*., 2013), trimethylamine (Wang *et al*., 2011), indole metabolites (Rothhammer *et al*., 2016; Alexeev *et al*., 2018), vitamins (LeBlanc *et al*., 2017), polyamines (Kibe *et al*., 2014) and secondary bile acids (Wahlström *et al*., 2016). These molecules play important roles to maintain good health and suppress onset of various diseases in the host. In order to obtain novel knowledge by combining the metabolome and microbiome analysis of the intestinal environment, a multi‐omics approach could be a valuable tool for understanding the entire intestinal ecosystem, including the relationships among microbiota, metabolites and host. Additionally, it is considered that a multi‐omics approach integrating metagenomics and metabolomics, called metabologenomics, can provide new information about the mechanisms underlying the key roles the microbiota plays in the varying conditions of the intestinal environment (Ishii *et al*., 2018).

Probiotics are live microorganisms that when consumed, exert beneficial effects on their hosts by either restoring imbalanced microbiota or maintaining the healthy microbiota population in the gut. Bifidobacteria and lactobacilli are the most common probiotics, and their biological and bacteriological properties have been studied extensively in order to characterize their beneficial functions (Ventura *et al*., 2008; Kleerebezem and Vaughan, 2009; Lebeer *et al*., 2010).

## General characteristics of bifidobacteria

In this short opinion, we would be placing our focus on bifidobacteria. Bifidobacteria are anaerobic and belong to the phylum Actinobacteria and the genus *Bifidobacterium*. They are Gram‐positive and are shaped like a letter Y. Representatives of this genus are present in a wide range of animal hosts and environmental sources but they are also reported to be present in fermented milk products and sewage (Milani *et al*., 2017). Human‐associated bifidobacteria are among the first colonizers and most abundant bacteria in the gut of infants who have been vaginally delivered and breastfed. Following weaning, the relative abundance decreases whilst their abundance further declines in elderly subjects (Tannock *et al*., 2016; Duranti *et al*., 2017). Bifidobacteria are highly regarded probiotics as their presence correlates with various health‐promoting activities and their absence has been reported to be related to health issues, such as obesity and undernutrition, especially with regard to infant gut microbiota (Arboleya *et al*., 2016). Bifidobacteria constitute part of the colonic gut community, and they contribute significantly to host metabolism via saccharolytic fermentation of glycans that are abundant in the proximal section of the colon (Rivière *et al*., 2016). The health‐promoting effects in the gut are attributed to the production of many metabolites such as vitamins, antioxidants, polyphenols, conjugated linoleic acids and SCFAs which have a positive impact on epithelial host cells as well as on the gut community (Hamer *et al*., 2012).

## Bifidobacteria and its protective mechanisms in the *E. coli* O157:H7 murine infection model

Enterohaemorrhagic *Escherichia coli* (EHEC) has been reported to be a cause for many illnesses ranging from mild diarrhoea to more severe diseases like haemolytic uraemic syndrome or haemorrhagic colitis (Tarr *et al*., 2005). EHEC O157:H7 produces Shiga toxin and is the major EHEC serotype involved in public health problems worldwide. Previous studies have shown previously that Shiga toxins 1 and 2 produced by *E. coli* O157 are pivotal factors in deadly infections (Eaton *et al*., 2008) and that if mice are pretreated with certain probiotics, including bifidobacteria, they survive with ideal outcomes. However, there is much to be understood about the protective mechanisms of bifidobacteria. In this report, we will review several articles on the protective mechanisms of bifidobacteria in the *E. coli* O157 murine infection model (Asahara *et al*., 2004; Gagnon *et al*., 2006; Yoshimura *et al*., 2009; Fukuda *et al*., 2011, 2012).

In a study by Yoshimura *et al*., the efficacy of bifidobacteria protection against *E. coli* O157:H7 infections was investigated using *E. coli* O157:H7‐infected gnotobiotic mice associated with *Bifidobacterium* strains (6 species, 9 strains). Survival rate in mice pre‐fed each *Bifidobacterium* strain for a week was recorded after they were orally infected with *E. coli* O157:H7. Mice gavaged with *Bifidobacterium longum* subsp*. infantis* 157F‐4‐1 and *B. longum* subsp*. longum* NCC2705 survived. On the other hand, mice associated with other *Bifidobacterium* strains, including type strains of *B. longum* subsp*. infantis* and *B. longum* subsp*. longum*, unfortunately perished. There were no significant differences in the numbers of *E. coli* O157:H7 in the faeces among the *Bifidobacterium*‐associated groups. However, in the gnotobiotic mice associated with *B. infantis* 157F‐4‐1 and *B. longum* NCC2705, they had significantly lower Shiga toxin concentrations in the caecal contents and sera than those of the other groups were observed. This experimental data the protection against the lethal infections of *E. coli* O157:H7 by *B. longum* subsp*. longum*/*infantis* could be due to prevention of caecal Shiga toxin production as well as Shiga toxin transfer from the intestinal lumen to the bloodstream (Yoshimura *et al*., 2009).

In another study using BALB/c mice, the efficacy of *Bifidobacterium thermacidophilum* RBL 71 as a probiotic against enterohaemorrhagic *E. coli* O157:H7 infection was studied. The mice were fed the probiotic for 7 days before or after a single challenge with *E. coli* O157:H7. In *B. thermacidophilum*‐treated mice, improvements in marked body weight loss and intestinal histopathological changes were observed as compared to the infected group. Pre‐feeding *B. thermacidophilum* RBL 71 for a week before infection resulted in larger food intake and increase in body weight, lesser extent of intestinal injuries, larger reaction in the lymphoid component of the ileal mucosa and lower faecal levels post *E. coli* O157:H7 challenge as compared to the infected controls. After infection, the concentrations of anti‐*E. coli* O157:H7‐specific faecal IgA and sera IgG + IgM were also increased in mice fed bifidobacteria. These results demonstrate that feeding the probiotic can alleviate the severity of *E. coli* O157:H7 infection (Gagnon *et al*., 2006).

In another report using the Shiga toxin‐producing *E. coli* O157:H7 mice model, Asahara *et al*. investigated the antiinfectious activity of probiotic bifidobacteria. *Bifidobacterium breve* strain was colonized in intestines of mice administered drinking water containing 5 mg ml^−1^ streptomycin. The infected controls had marked body weight loss and subsequently died, but this was significantly inhibited in the *B. breve*‐colonized group. Moreover, Shiga toxin production by intestinal cells was almost undetectable in the *B. breve*‐colonized group. Several *Bifidobacterium* strains that were naturally resistant towards streptomycin were compared for anti‐Shiga toxin‐producing activity. In the potent strains such as *B. breve* strain Yakult and *Bifidobacterium pseudocatenulatum* DSM 20439, higher concentration of acetic acid of approximately 56 mM and lower intestinal pH were observed as compared to the infected control group (acetic acid concentration, 28 mM; pH, 7.15). Both high concentrations of acetic acid and a low pH due to these bifidobacterial strains could impede Shiga toxin production *in vitro* and these characteristics contributed to the protective effect against the lethal infection (Asahara *et al*., 2004).

In our previous studies, we also reported that acetate produced by certain bifidobacterial strains in mice associated with bifidobacteria was protected against enterohaemorrhagic *E. coli* O157:H7 using a multi‐omics approach. Certain bifidobacterial possess an ATP‐binding‐cassette‐type carbohydrate transporter, thereby increasing the production of acetate and inhibited translocation of *E. coli* O157:H7 produced Shiga toxin from the gut lumen to the blood. The protective bifidobacteria produced acetate, thereby upregulating intestinal defence mediated by epithelial cells and protected the host against lethal infection (Fukuda *et al*., 2011, 2012).

## Conclusions

**Figure 1:**
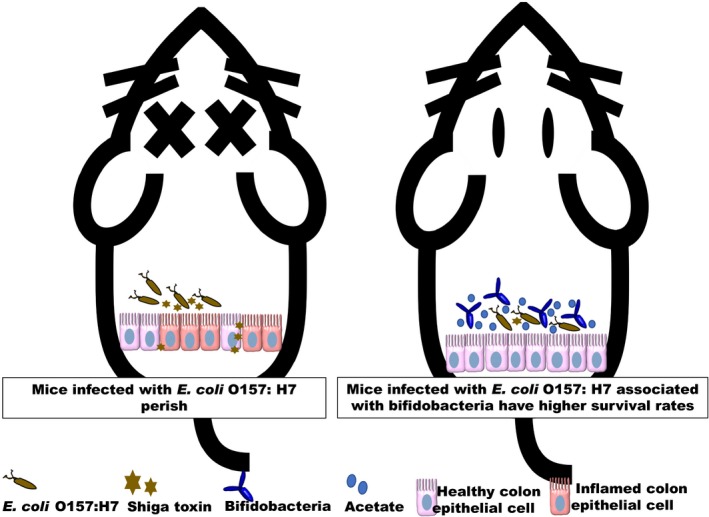
Mice infected with *E. coli* O157: H7 associated with bifidobacteria have higher survival rates as some bifidobacterial strains possess an ATP‐binding‐cassette‐type carbohydrate transporter, thereby increasing the production of acetate and inhibited translocation of *E. coli* O157:H7 produced Shiga toxin from the gut lumen to the blood.

Lately, there has been overwhelming developments in identifying bifidobacterial structures that play important roles in host colonization and exert health‐promoting effects on the host. In this short opinion article, we have chosen to focus on summarizing the protective effects of bifidobacteria and its metabolite: acetate; against enteropathogens, specifically in the *E. coli* O157:H7 mice model (Fig. 1). There is much more to be elucidated, and more contributions to the scientific field could be made possible by integrating multi‐omics evaluations using various disease murine models or clinical subjects.

## Conflicts of interest

The authors declare no conflict of interest.
